# Augmented renal clearance in the ICU: estimation, incidence, risk factors and consequences—a retrospective observational study

**DOI:** 10.1186/s13613-022-01058-w

**Published:** 2022-09-26

**Authors:** Alexandre Egea, Claire Dupuis, Etienne de Montmollin, Paul-Henry Wicky, Juliette Patrier, Pierre Jaquet, Lucie Lefèvre, Fabrice Sinnah, Mehdi Marzouk, Romain Sonneville, Lila Bouadma, Bertrand Souweine, Jean-François Timsit

**Affiliations:** 1 Service d’Anesthésie Réanimation, CHU Saint Antoine, APHP, Paris, France; 2 Service de Médecine Intensive et Réanimation, CHU Clermont Ferrand, CHU Hôpital Gabriel-Montpied, 58 Rue Montalembert, 63000 Clermont Ferrand, France; 3 Medical and Infectious Intensive Care Unit, CHU Bichat-Claude, APHP, Paris, France; 4 IAME UMR 1137, Université de Paris, 75018 Paris, France; 5 Service de Médecine Intensive-Réanimation, iCAN, Institut de Cardiologie, Sorbonne Université Hôpital Pitié-Salpêtrière, APHP, Paris, France; 6 Réanimation Polyvalente/Surveillance Continue, Hôpitaux Publics de l’Artois, Lens, France; 7 Université de Paris, UMR1148, Team 6, 75018 Paris, France

**Keywords:** Augmented renal clearance, Intensive care unit, Epidemiology

## Abstract

**Background:**

Augmented renal clearance (ARC) remains poorly evaluated in ICU. The objective of this study is to provide a full description of ARC in ICU including prevalence, evolution profile, risk factors and outcomes.

**Methods:**

This was a retrospective, single-center, observational study. All the patients older than 18 years admitted for the first time in Medical ICU, Bichat, University Hospital, APHP, France, between January 1, 2017, and November 31, 2020 and included into the Outcomerea database with an ICU length of stay longer than 72 h were included. Patients with chronic kidney disease were excluded. Glomerular filtration rate was estimated each day during ICU stay using the measured creatinine renal clearance (CrCl). Augmented renal clearance (ARC) was defined as a 24 h CrCl greater than 130 ml/min/m^2^.

**Results:**

312 patients were included, with a median age of 62.7 years [51.4; 71.8], 106(31.9%) had chronic cardiovascular disease. The main reason for admission was acute respiratory failure (184(59%)) and 196(62.8%) patients had SARS-COV2. The median value for SAPS II score was 32[24; 42.5]; 146(44%) and 154(46.4%) patients were under vasopressors and invasive mechanical ventilation, respectively. The overall prevalence of ARC was 24.6% with a peak prevalence on Day 5 of ICU stay. The risk factors for the occurrence of ARC were young age and absence of cardiovascular comorbidities. The persistence of ARC during more than 10% of the time spent in ICU was significantly associated with a lower risk of death at Day 30.

**Conclusion:**

ARC is a frequent phenomenon in the ICU with an increased incidence during the first week of ICU stay. Further studies are needed to assess its impact on patient prognosis.

**Supplementary Information:**

The online version contains supplementary material available at 10.1186/s13613-022-01058-w.

## Background

Augmented renal clearance (ARC) is most of the time defined by a glomerular filtration rate (GFR) above 130 mL/min/1.73 m^2^ [1]. The pathophysiology of ARC is generally difficult to elucidate and could be secondary to several factors in intensive care (ICU) including 1. Increase of cardiac output and therefore renal blood flow [2–4], mostly in case of inflammatory response syndrome; 2. Mobilization of the functional nephron reserve, mostly in younger and/or obese patients [5]; or 3. Endocrine responses including atrial natriuretic peptide [6].

Few studies focused on ARC in ICU. The prevalence of ARC was between 20 and 65% [7], and was higher in the sub groups of patient with cerebro-vascular disorders [8] or severe burns [9]. The main factors of ARC were age [10–14], reason for admission (trauma) [2, 11, 14–16] and severity on admission [2, 15–17]. ARC was also associated with under antibiotic dosage including vancomycin [13, 16, 18–20] and beta-lactams [21–26]. However, the relationship between ARC and therapeutic failure or prognosis is less clear and not well described [21, 24, 27–29].

Unfortunately, those studies are scarce and most of the time based on small and specific cohort of critically ill patients.

Furthermore, very few studies focused on the evolution profile of ARC. Fuster-lluch et al. reported an incidence of ARC of 17.9% on admission which increased to 30% during the first week of ICU stay [17]. Similar results were found by Udy et al. which also underlined that most of the ARC occurred during the first week after ICU admission [11]. Moreover, De Waele et al. underlined that only 30% of the patients remained with ARC during their whole ICU stay [12].

In that context, the aim of our study was to describe the evolutionary profile of ARC in ICU, to determine the factors associated with the occurrence of early and secondary ARC and to assess the impact of ARC on ICU death.

## Methods

### Type of study, population, and data source

We conducted a retrospective, monocentric, observational study including patients admitted for the first time to the medical and infectious resuscitation department of Bichat-Claude-Bernard University Hospital, APHP, France, between January 1, 2017 and November 31, 2020 and included in the OutcomeRea database. Patients were excluded if they had dialysis-dependent chronic kidney disease (CKD) as defined by the Knaus Scale [30], if their length of stay (LOS) was less than 72 h, if serum creatinine was never measured during their ICU stay or if GFR could not be measured during the whole ICU stay.

### Ethical considerations

Outcomerea is an observational prospective multicenter cohort. The clinical and biologic data of each included patients are registered in the database each day of the ICU stay. This database has been approved by the French Advisory Committee for Data Processing in Health Research (CCTIRS), by the Institutional Review Board (CECIC Clermont-Ferrand -IRB n°5891; Ref: 2007-16) and the French Informatics and Liberty Commission (CNIL) which waived the need for signed informed consent of the participants, in accordance with French legislation on non-interventional studies. This study did not require individual patient consent because it involved research on a previously approved database.

### Collected data and definition

The GFR was estimated using the measured Creatinine Renal Clearance (CrCl) based on 24-h urine output, corresponding creatinine and urinary creatinine (Additional file 1: Table S1).

ARC was defined by a GFR above 130 mL/min/1.73 m^2^.

Patients on renal replacement therapy had a zero GFR from the beginning to the end of the last renal replacement therapy session during their stay in the ICU.

GFR was estimated from day 1 to day 30 in ICU.

Patients were considered to have ‘ARC on admission’ if they had ARC on day 1 or day 2 of ICU stay. Patients who had ARC for the first time after Day 2, were considered to have ‘late ARC’.

Acute kidney injury (AKI) was defined using the KDIGO (Kidney Disease: Improving Global Outcome) [31] classification using serum creatinine item and urinary output (UO). However, since our database does not track UO at 6 h, but only for 24 h, one cannot distinguish between the subgroups of AKI (KDIGO stage 1 and 2). Under the assumption that the requirement of the UO to be less than 0.5 mg/kg/h for 24 h is more stringent than for 6 h, we assigned all patients with UO less than 0.5 ml/kg/h to KDIGO stage 2 [32].

Baseline serum creatinine levels were measured in blood samples taken before hospital admission when available. In times the baseline creatinine level or GFR was not available, the lowest serum creatinine level measured during the patient’s hospital stay was used if the GFR was ≥ 75 ml/min/1.73 m2. In other cases, the baseline creatinine level was estimated by using the Modification of Diet in Renal Disease equation with a normal GFR value of 75 ml/min/ 1.73 m^2^ [33].

Severe COVID-19 pneumonia was defined as the combination of: (1) radiological features compatible with this diagnosis; (2) PaO2/Fio2 ratio ≤ 300 mm Hg, and (3) a positive severe acute respiratory syndrome coronavirus-2 test using reverse transcriptase polymerase chain reaction.

### Data collection

The following data were collected at admission: age, sex, reason for admission, comorbidities as defined by the Knaus Scale [30], severity of illness at ICU admission (SAPS II, SOFA scores), main symptoms on admission, organ failures and organ support including vasopressors, renal replacement therapy, invasive mechanical ventilation and other treatments including the use of parenteral or enteral nutrition, vancomycin, aminoglycoside or proton-pump inhibitors and the administration of iodinated contrast media during CT scans.

Outcomes recorded were ICU and hospital LOS, vital status at the end of ICU and hospital stay.

### Outcomes and subgroup analyses

Main outcomes were the occurrence of ARC on admission and late ARC and ICU death.

### Statistical analyses

The data were described as mean and standard deviation or median and interquartile range for continuous data according to their distribution, and the categorical data as number and percentage. Comparisons relied on the Fisher exact test for categorical data and Wilcoxon test for continuous data.

Missing data were imputed linearly. GFR were first measured using complete data and then linearly imputed. Daily distributions of the missing data are reported in Additional file 1: Table S2.

The cumulative incidence curve over time of the first episode of ARC was plotted.

Univariate and then multivariate logistic regression analysis were performed to determine factors associated with the presence of ARC at admission. Variables achieving a *p*-value < 0.2 were tested in the multivariate model. Variables were selected by forward–backward analysis to define the final model. Only variables with a *p*-value < 0.05 were retained in the final model.

The factors associated with the occurrence of ‘late ARC’ were sought by a Fine Gray-type subdistribution survival model, taking death or alive discharge from the ICU as the competing risk. A univariate and then multivariate analysis were conducted in a similar manner with univariate variable selection and forward/backward selection for the final model.

Finally, we reported the cumulative probability of death overtime according to admission status: ARC, No ARC/No AKI; AKI KDIGO 1–2 and AKI KDIGO 3. Subgroup analyses were achieved among the patients with SARS-COV 2 and under anti-microbial therapy.

A logistic regression model evaluating the association between the occurrence of death and the "% of stay with ARC" adjusted to the LOS was performed. As sensitive analyses, cause-specific models with death and being discharged alive from ICU as outcomes considering ARC as a time-dependent covariate during ICU stay were achieved with a landmark approach from Day 2 to Day 7. Sub-distribution survival analyses considering being discharged alive from ICU as competing risk and assessing the effect of ARC on Day 2 among the patients still alive on Day 2, the effect of ARC on Day 3 among the patients still alive on Day 3, and so on until Day 7 were also achieved.

All analyses were performed with SAS software version 9.4 (SAS Institute Inc., Cary, North Carolina) or R software with the "mstate" library.

## Results

### Baseline characteristics

During the period of the study, 1313 were admitted in ICU but only 784 patients were recorded in the Outcomerea data base. Only 312 patients were included in the study (Additional file 1: Fig. S1). Main characteristics of the patients are presented in Additional file 1: Table S3. The median age was 62.7 years [51.3; 71.8], 75% of the patients were male, 26.9 of them were obese, the main comorbidities were cardiovascular (31.9%), respiratory (19.3%) and immunosuppression (18.7%). The main reason for admission was respiratory failure (59%) and shock (13%) including 62.8% patients with COVID-19 pneumonia. The SAPS II score was 32 [24; 43]. Patients on admission required treatment with vasopressor, mechanical ventilation, and extra renal replacement therapy in, respectively, 44%, 46.4% and 14.5% of cases. The median LOS in the ICU was 9 days [5; 17] with 31% of deaths in the ICU.

### ARC prevalence, incidence, and outcomes

**Fig. 1:**
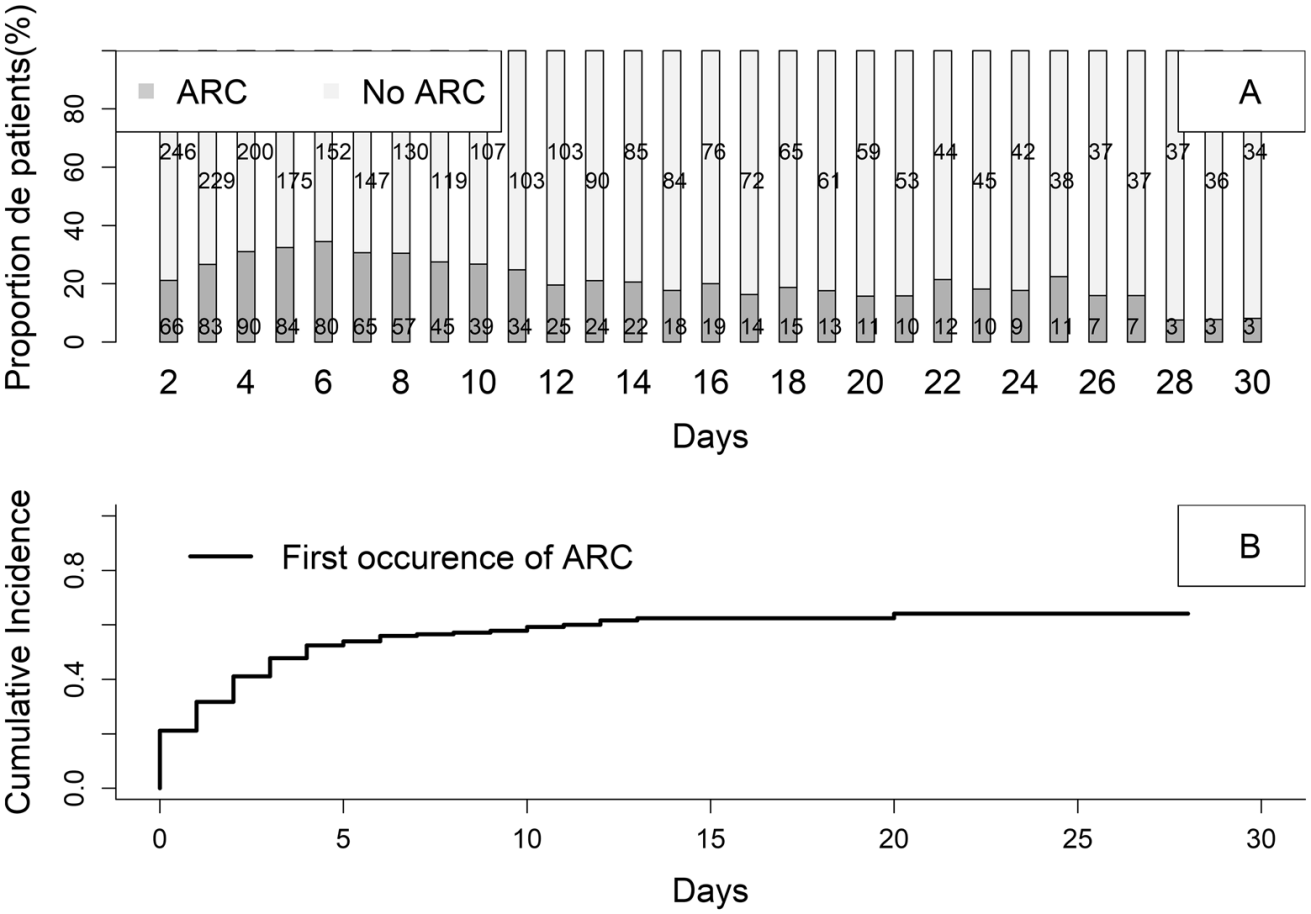
Daily prevalence (**A**) and cumulative incidence (**B**) of augmented renal clearance in ICU from day 1 to day 30

The median daily prevalence of ARC during ICU stay was 24.6%. At Day 2, the prevalence of ARC was 21%. The maximum was reached at Day 6 (34.4%) and then decreased from Day 7 until Day 12, to remain stable afterwards at around 20% (Fig. 1A). The cumulative incidence rate was around 60% at Day 7 (Fig. 1B). Concerning the evolution of GFR according to renal function at admission, patients with ARC stayed with high GFR during hospitalization. Patients without AKI and without ARC, but also patients with AKI KDIGO 1–2 presented a progressive increase in GFR up to the 5th day of hospitalization. The GFR then decreased over time. Most of the patients with AKI KDIGO 3 kept their renal failure during the whole ICU stay. Of note, only patients with KDIGO CKD Stage 1 to 3 presented ARC during their ICU stay, but none of the patients with KDIGO CKD stage 4 on admission (Additional file 1: Table S4).

### Risk factors and ARC on admission and late ARC

**Fig. 2:**
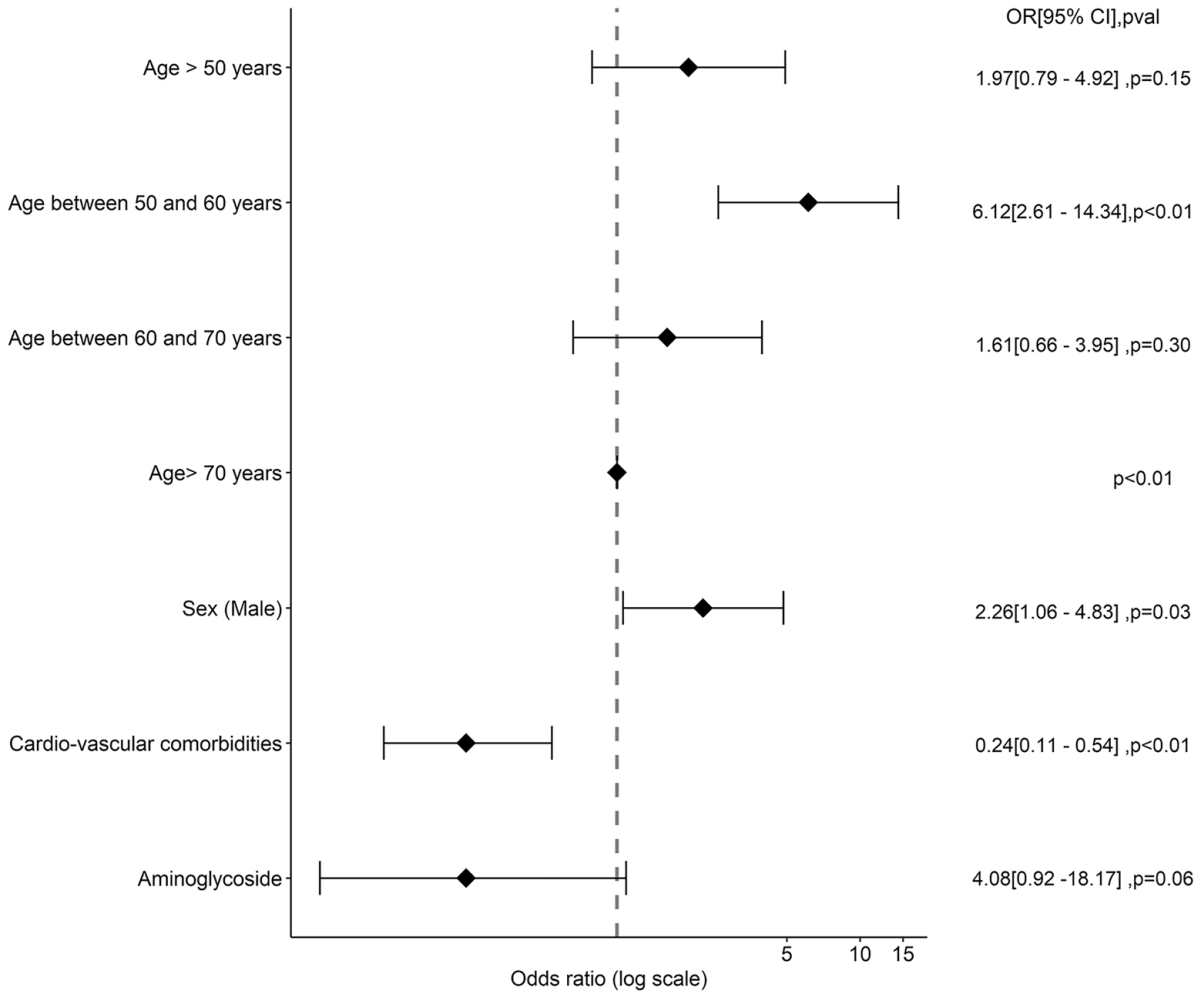
Factors associated with ARC at admission. *OR* odds ratio, *CI* 95% confidence interval, *HR* hazard ratio. Factors tested in multivariate analysis for the of ARC at admission were age, sex (male), cardiovascular comorbidities, immunosuppression, diabetes, SARS-COV2, catecholamines, invasive mechanical ventilation, proton-pump inhibitors, enteral nutrition, aminoglycosides

**Fig. 3:**
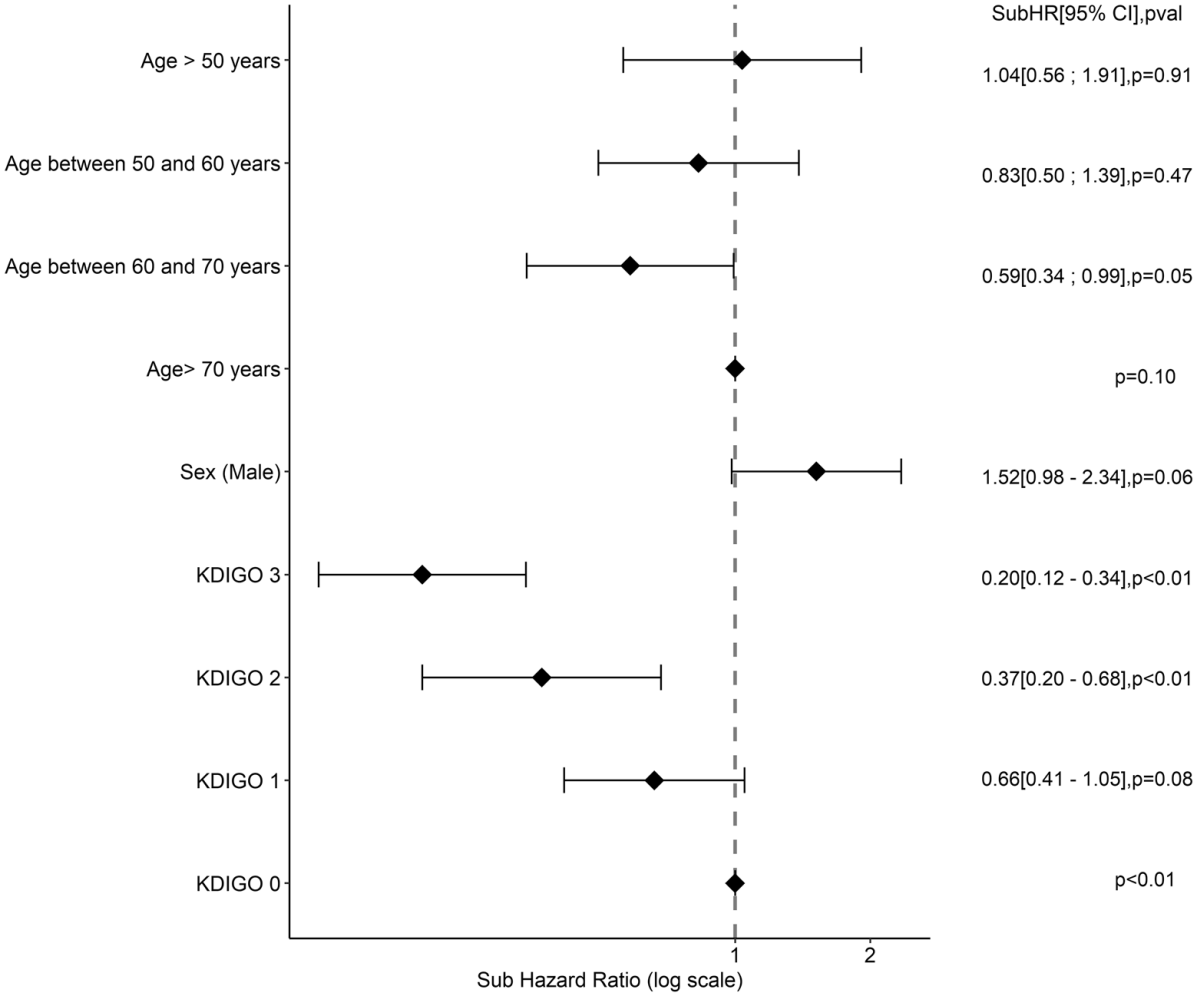
Factors associated with late ARC—multivariate analyses. *OR* odds ratio, *CI* 95% confidence interval, *HR* hazard ratio. Factors tested in multivariate analysis for the risk of late ARC were age, sex (male), BMI > 30 kg/m^2^, SARS COV 2 pneumoniae, immunosuppression, catecholamines, KDIGO (Kidney Disease: Improving Global Outcome score), parenteral nutrition, aminoglycosides, vancomycins, proton-pump inhibitors

Comparisons of the patients with ARC and no ARC at admission are reported in Table S3. Patients with ARC at admission were younger (55 years [50.2; 63.5] vs. 64 years [53.1; 73], *p* < 0.01), with fewer cardiovascular comorbidities and diabetes. They were less severe (SAPS II: 24 [20; 34] vs 34 [26; 45]; *p* < 0.01); had less renal failure and were less exposed to aminoglycosides. In multivariable analysis, a younger age, absence of cardiovascular comorbidities and absence of exposition to aminoglycosides on admission were associated with the occurrence of ARC at admission (Fig. 2, Additional file 1: Table S5). The absence of renal failure on admission was associated with late ARC (Fig. 3, Additional file 1: Table S6).

### ARC and mortality in ICU

Risk factors for mortality before Day 30 are shown in Additional file 1: Table S7. The survivors at Day 30 had a higher rate of ARC at admission than non-survivors (54(25.5%) vs 12(12%), *p*-value < 0.01), and spent more time with ARC during their ICU stay (%time with ARC 16.7%[0; 50]) versus 0%[0; 18.2], *p* < 0.01).

**Fig. 4:**
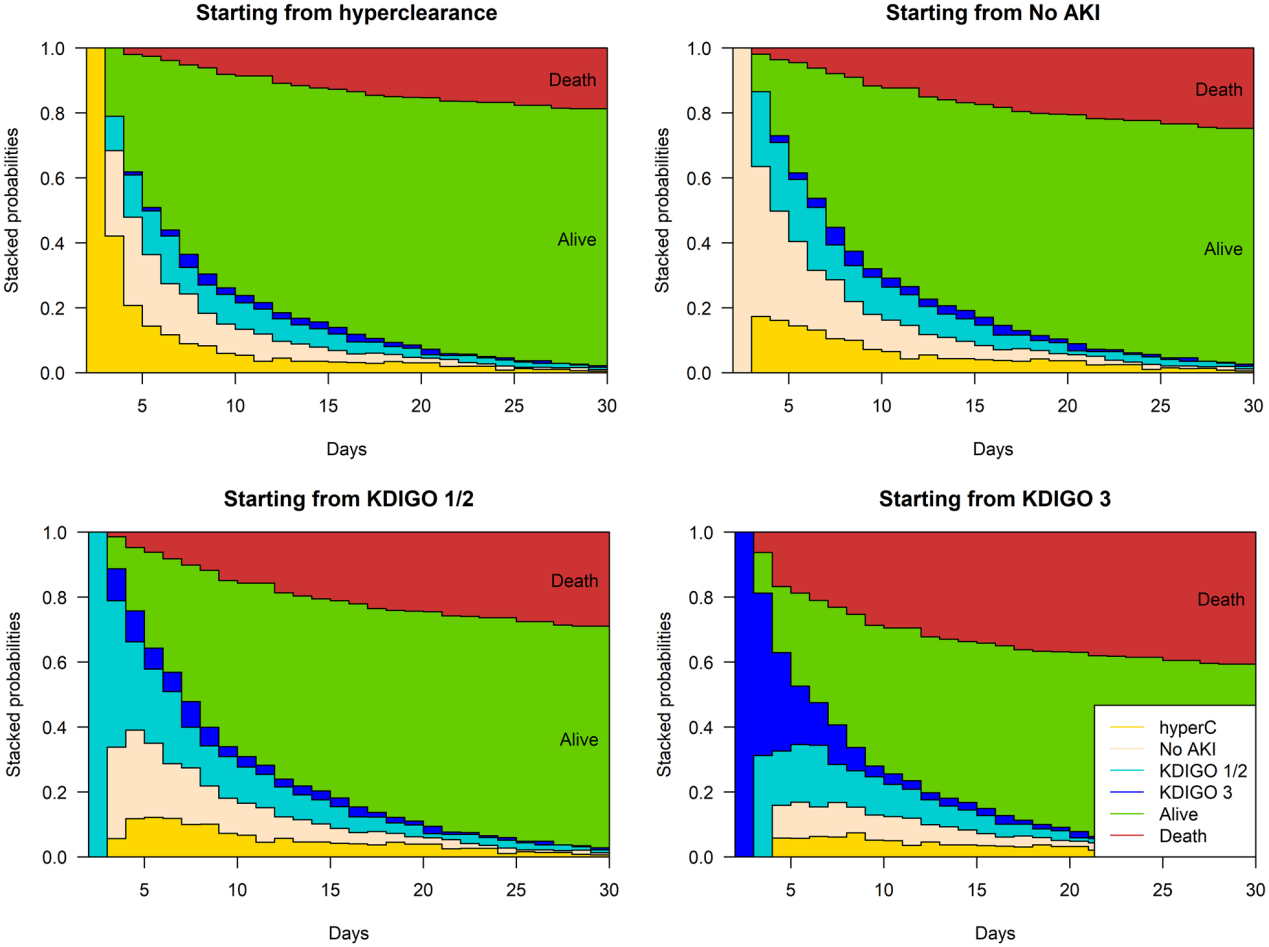
Cumulative probability of being at a state at a given time after the ICU stay for patients with (from left to right) ARC at admission, without renal failure or ARC, with KDIGO (KDIGO (Kidney Disease: Improving Global Outcome score) 1 or 2 renal failure, and with KDIGO 3 renal failure at admission

The probability of death during the ICU stay differed according to the renal admission status of the patient (Fig. 4, Additional file 1: Table S8). Patients with ARC at admission had a better survival compared to patients with normal renal function on admission (79% vs. 73%). Patients with normal renal function on admission also had a better probability of survival over time compared to patients with AKI on admission (68% KDIGO 1|2 and 57% for KDIGO 3).

**Fig. 5:**
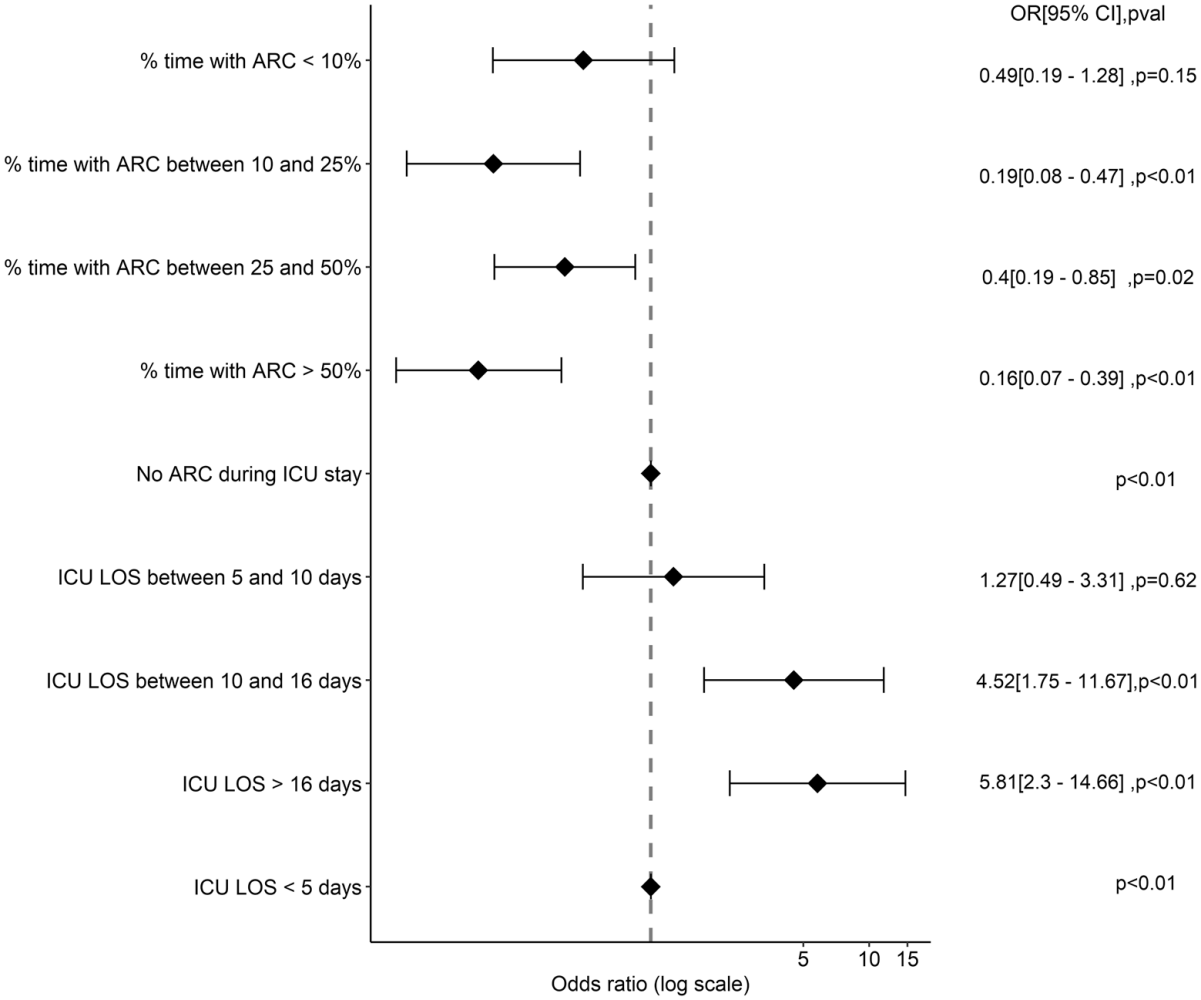
Association between the percentage of time with ARC (augmented renal clearance) during ICU (intensive care unit) stay and ICU mortality (multivariate logistic regression model), LOS (length of stay)

Finally, a time spent with an ARC > 10% of the ICU stay prevented from death (between 10 and 25%: OR = 0.19 [0.08–0.47], *p* < 0.01; between 25 and 50%: OR = 0.4 [0.19–0.85], *p* = 0.01; > 50%: OR = 0.16 [0.07–0.39], *p* < 0.01) (Fig. 5). The sensitive analyses using cause-specific survival models and subdistribution survival models considering being discharged alive from ICU as a competing risk and using a landmark approach from Day 2 to Day 7 also found a protective effect of ARC on death (Additional file 1: Table S9 and S10).

In the subgroup of patients with SARS-COV2 on admission or anti-microbial therapy, the time spent with ARC during ICU stay was similarly associated with death at Day 28 (data not shown).

## Discussion

We found a high overall prevalence of ARC at 24.6%, with an increase of the incidence from the admission to Day 6 of ICU stay. The protective factors associated with the occurrence of ARC on admission were young age and low severity on admission. Patients with cardiovascular comorbidities and AKI on admission (KDIGO 3) had less chance of developing ARC during the stay. Finally, ARC and its persistence during the ICU stay was associated with a better patient prognosis.

These results deserve some comments.

First, we found a relatively high prevalence of ARC in ICU, which was already reported [7], highlighting the need to monitor GFR. However, it is interesting to notice that the prevalence observed in the literature changed with the methods used to estimate GFR. Indeed, studies evaluating GFR using equations based on serum creatinine often found lower prevalence of ARC (less than 10%), because of the underestimation of GFR by these formulas [29, 34]. In addition, prevalence varies also according to the inclusion criteria of the studies (e.g., exclusion of patients with AKI at admission), or definitions of ARC [6, 17].

Second, we observed a "rebound" of ARC between Day 5 and Day 7, mostly for the patients with normal renal function or AKI KDIGO 1 or 2. Several studies have already reported this “rebound” effect. Udy et al. showed that 65% of critically ill patients had at least one episode of ARC, and that patients with ARC on admission tended to remain with ARC during their ICU stay. They underlined also that the highest clearance value was most of the time reached at Day 5 [11]. Fuster-lluch et al. also found that most of the ARC were reached at Day 5 with a 30% prevalence of ARC at Day 5 [17]. Similar results were found by Brown et al. in a cohort of polytraumatized patients [4]. The main explanation for this "rebound" effect could be the implementation of compensatory and recovery mechanisms by the organism in response to the administration of fluid therapy and vasopressors to improve cardiac output and renal perfusion [3]. Our study thus underlines the need to monitor every day renal clearance, with a particular interest for patients without renal failure or with rapidly reversible AKI.

Third, we identified young age and the absence of cardiovascular comorbidities as factors associated with ARC on admission in multivariate analysis. Young age is the main factor associated with ARC in the literature. The combination of a systemic inflammatory response syndrome and a higher renal functional reserve observed in younger patients may explain why these patients have more chance of developing ARC. The absence of cardiovascular comorbidities on admission has not to our knowledge been reported as a factor associated with ARC. Cardiovascular pathologies being associated with a risk of chronic kidney disease, this factor seems logically associated with the absence of ARC. Then, we are the first to report factors associated with secondary ARC occurrence, that is absence of KDIGO 2–3 AKI. This result agrees with the rebound of GFR observed in patients without AKI or with KDIGO 1 AKI, highlighting once again the importance of close monitoring of renal clearance in these patients during the whole ICU stay. Moreover, these results highlight that patients with ARC have a better renal functional reserve explaining also the association found between ARC and young age.

At last, we showed that ARC and its persistence during ICU stay were associated with a better prognosis. Several studies have investigated the association between ARC and poor prognosis and therapeutic failures related to underdosing of antibiotics in ICU. Most of them were negative [21, 28, 29, 34]. One explanation could be that ARC is also an organ failure recovery marker, and therefore associated with better outcome. Mulder et al. found in their study an association between ARC and a decrease in mortality in a trauma unit, hypothesizing that ARC is a beneficial compensation mechanism for trauma [35]. However, it is difficult to establish a relationship between prognosis and ARC in the ICU. ARC is both a marker of good renal functional reserve and may be responsible for antibiotic underdosing and thus therapeutic failure. The relationship between ARC and prognosis is therefore complex and deserves to be studied in more detail. In our study, only a part of the patients was admitted for sepsis and received antibiotics at admission. In addition, drug dosing especially antibiotics are adapted according to the estimated glomerular filtration rate. In case of ARC antibiotic dosing is increased. It is therefore very likely that the prognosis observed in relation to ARC is primarily a reflection of the severity of the patients and their young age.

Our study has several limitations. First, it is a retrospective, single-center study, limiting the internal validity of our results. Second, the population is mainly medical patients, with 18.9% of patients of the immunocompromised cohort and a significant proportion of patients hospitalized for SARS-COV-2 pneumonia, which limits the external validity of our results. Third, several variables of interest were not recorded including diuretic use, calory and protein intakes and fluid balance, preventing from time-dependant analyses. Another important issue concerns the GFR measurement technique. CrCl overestimates GFR, due to tubular secretion of creatinine, but remains well correlated with inulin clearance [36]. Furthermore, collection of urine over 24 h may be difficult, with a risk of urine loss. In this context, some authors suggest measuring GFR over shorter periods [37, 38]. There is still, however, no clear consensus on the duration of the urine collection period to obtain a more accurate estimate of GFR. Other exogenous markers can also be used to better characterize ARC, in particular the use of iodinated contrast agent. Sangla et al. have found a low precision and an important bias of CrCl compared with plasma iohexol clearance probably due to imprecision in urine collection, sarcopenia and renal secretion of creatinine [39]. Although there are different techniques for estimating GFR, the calculation of urinary creatinine clearance remains the main technique for assessing GFR in ICU due to its feasibility and low cost. Then, we could not precisely distinguish patient with KDIGO 1 from patient with KDIGO 2 AKI because our database does not track UO at 6 h or 12 h. Lastly, missing data concerning GFR during ICU stay might have biased our results. However, most of the GFR missing were related to missing urinary creatine, with low urinary output and therefore considered with low GFR.

This work opens several perspectives, including studies focusing on patients without AKI or only AKI KDIGO 1 or 2 on admission, i.e., those at risk of ARC. More reliable measurements of GFR by exogenous markers combined with cardiac output, renal perfusion and fluid balance measurements and data on diuretics intakes would allow better characterization of the evolutionary profile of ARC in the ICU. The study of antibiotic dosage in the presence of ARC and the impact of their adaptation would also deserve a prospective work.

## Conclusion

In conclusion, one-quarter of our patients had an episode of ARC in the ICU. To detect ARC and adapt anti-microbial therapy, a daily GFR measurement should therefore mostly be performed for the ICU patients under anti-microbial therapy with a particular attention for younger patients without ARC on admission, with a normal or moderately impaired renal function. ARC is a common phenomenon in the ICU, but its pathophysiology and impact on patient outcomes are still poorly understood. Further studies are needed to better understand the evolutionary profile of ARC and its implication in the management of ICU patients.

## Supplementary Information

**Additional file 1.** Additional tables and figures.

## Data Availability

Data are available upon request from the corresponding author.

## Abbreviations

ARC: Augmented renal clearance
AKI: Acute kidney injury
CCTIRS: Committee for Data Processing in Health Research
CKD: Chronic kidney disease
CNIL: French informatics and liberty commission
CrCl: Measured creatinine renal clearance
GFR: Glomerular filtration rate
ICU: Intensive care unit
KDIGO: Kidney Disease: Improving Global Outcome
LOS: Length of stay
OR: Odds ratio
SOFA: Sequential Organ Failure Assessment
SAPS: Simplified Acute Physiology Score
UO: Urine output
