# Supplementary material for: Augmented renal clearance in the ICU: estimation, incidence, risk factors and consequences—a retrospective observational study
Source: Ann Intensive Care. 2022 Sep 26;12:88. doi: 10.1186/s13613-022-01058-w (PMC9510087; doi:10.1186/s13613-022-01058-w)
Supplement: Supplementary file 1 — Additional file 1. Additional tables and figures. [file 13613_2022_1058_MOESM1_ESM.docx]

**Supplementary data**

Table S 1: Equations used for creatine clearance and the Body Surface Area

| Formulas |
| --- |
| ClCr (ml/min/1,73m²) = (U x V x 1000 / P x 1440) x 1,73/SC |
| BSA (Body Surface Area) = 0.007184 × H^0.725^ × W^0.425^ |

BSA, Body Surface Area (m²); ClCr, Creatinine Clearance; H, Height (cm²) ; P, Plasmatic creatinine concentration (µmol/ml); U, Urinary creatinine concentration (mmol/ml)


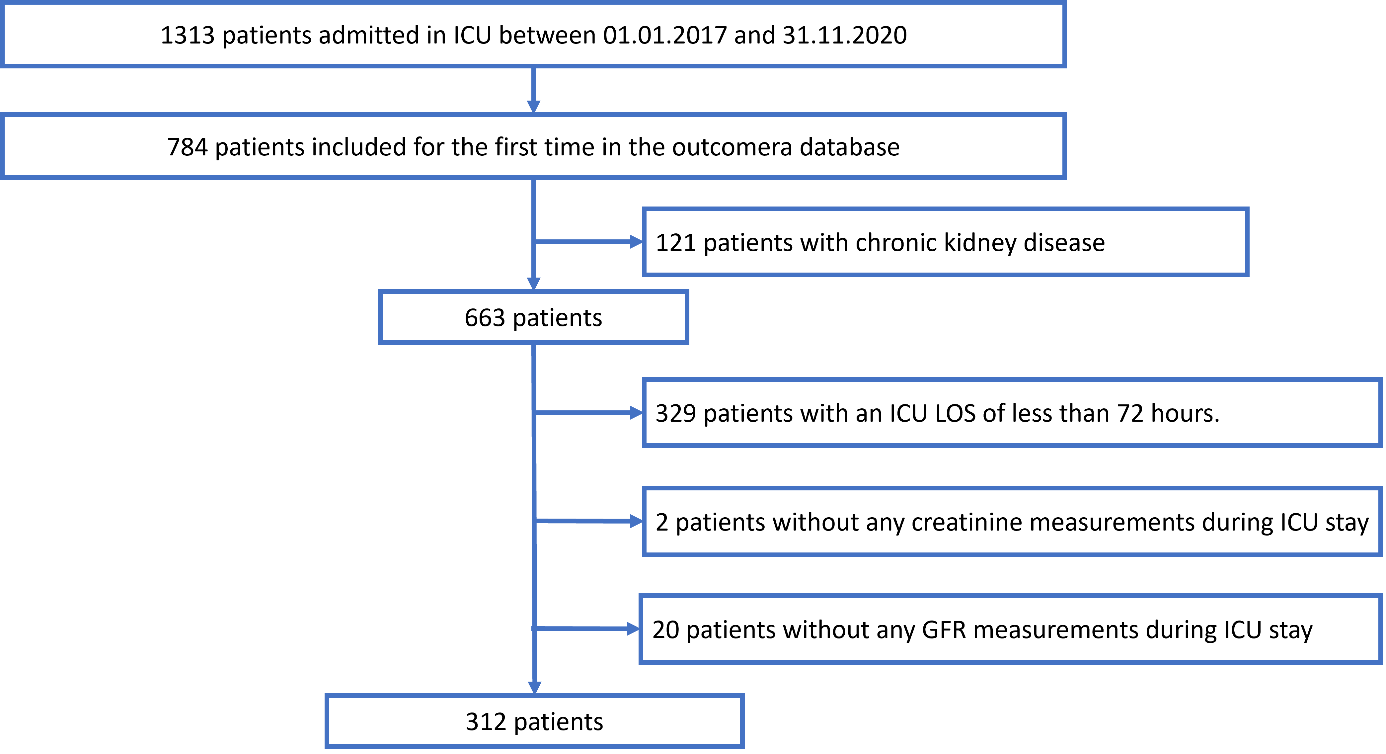


Figure S1: Flow chart

ICU: intensive care unit; LOS: Length of stay; GFR: glomerular filtration rate

Table S 2: Daily distribution of missing data for creatinine, urinary output, urinary creatinine, and GFR from Day 2 to Day 30 in ICU

| Day | Numbers of patients | Creatinine | miss | Urinary output | miss | Urinary creatinine | miss | Calculated GFR | miss |
| --- | --- | --- | --- | --- | --- | --- | --- | --- | --- |
| 2 | 312 | 85 [61 ; 134] | 7 | 1.1 [0.7 ; 1.7] | 3 | 7 [4.4 ; 10.4] | 88 | 56.2 [17.9 ; 102.8] | 74 |
| 3 | 312 | 83 [59 ; 136] | 4 | 1.1 [0.7 ; 1.7] | 3 | 8.6 [5.4 ; 11.7] | 81 | 76.1 [16.9 ; 135.7] | 65 |
| 4 | 290 | 86 [57.5 ; 136.5] | 10 | 1.3 [0.7 ; 1.8] | 5 | 8.2 [5.6 ; 11.9] | 80 | 77 [18.4 ; 144.7] | 67 |
| 5 | 259 | 81 [58 ; 149] | 4 | 1.3 [0.7 ; 2] | 10 | 7.9 [5.3 ; 11.9] | 74 | 76.9 [14.2 ; 156.1] | 57 |
| 6 | 232 | 84 [58 ; 170] | 7 | 1.3 [0.7 ; 2.1] | 5 | 8 [5.1 ; 10.8] | 71 | 74.9 [8.7 ; 167.3] | 50 |
| 7 | 212 | 91 [62 ; 186] | 10 | 1.3 [0.6 ; 2] | 9 | 7.2 [4.2 ; 10.4] | 79 | 62.2 [0 ; 119.2] | 58 |
| 8 | 187 | 95.5 [61 ; 197] | 9 | 1.3 [0.4 ; 2] | 9 | 7.2 [4.6 ; 10.4] | 61 | 59.5 [0 ; 144.1] | 38 |
| 9 | 164 | 99 [58 ; 205] | 7 | 1.3 [0.2 ; 2] | 8 | 7.5 [5.2 ; 10.3] | 55 | 57.9 [0 ; 128] | 30 |
| 10 | 146 | 93 [58 ; 183] | 4 | 1.2 [0.2 ; 1.9] | 5 | 6.6 [4.5 ; 9.7] | 66 | 40.3 [0 ; 103.4] | 38 |
| 11 | 137 | 95 [64 ; 201] | 8 | 1.1 [0.3 ; 1.8] | 5 | 7.6 [4.2 ; 10.8] | 52 | 47 [0 ; 96.3] | 28 |
| 12 | 128 | 104 [62 ; 202] | 6 | 1 [0.2 ; 2] | 7 | 6.5 [3.2 ; 10.4] | 57 | 23.8 [0 ; 93.6] | 27 |
| 13 | 114 | 103 [58 ; 214] | 7 | 1.2 [0.1 ; 2] | 6 | 6.7 [3.3 ; 10.1] | 40 | 40.7 [0 ; 108.6] | 16 |
| 14 | 107 | 102 [61 ; 197] | 4 | 1.2 [0.2 ; 1.8] | 4 | 7.8 [3.1 ; 10.2] | 45 | 9.5 [0 ; 99.2] | 18 |
| 15 | 102 | 108 [65 ; 192] | 5 | 1.2 [0.1 ; 2] | 3 | 6.8 [3.3 ; 9.7] | 50 | 0 [0 ; 91.6] | 19 |
| 16 | 95 | 128 [64 ; 206] | 8 | 1.2 [0.1 ; 2] | 3 | 5.9 [2.8 ; 9.7] | 37 | 1.7 [0 ; 98.1] | 15 |
| 17 | 86 | 122 [61 ; 197] | 6 | 1.1 [0.1 ; 1.9] | 5 | 5.7 [2.4 ; 8.3] | 40 | 0 [0 ; 73.9] | 14 |
| 18 | 80 | 125 [62 ; 209] | 6 | 1.1 [0.1 ; 1.8] | 2 | 6.5 [2.7 ; 9.6] | 37 | 0 [0 ; 105.4] | 13 |
| 19 | 74 | 113 [63 ; 205] | 3 | 0.9 [0.1 ; 1.9] | 2 | 6.2 [3.7 ; 9.1] | 39 | 0 [0 ; 83.3] | 14 |
| 20 | 70 | 119.5 [61 ; 204] | 4 | 1 [0.1 ; 1.6] | 3 | 5.4 [3.1 ; 9.7] | 31 | 0 [0 ; 78.5] | 11 |
| 21 | 63 | 114.5 [59.5 ; 184.5] | 3 | 0.9 [0 ; 1.6] | 0 | 5 [2.2 ; 8.7] | 32 | 0 [0 ; 74.2] | 14 |
| 22 | 56 | 118.5 [58 ; 183] | 2 | 1.2 [0.1 ; 1.9] | 0 | 6 [2.8 ; 9.5] | 27 | 0 [0 ; 108.5] | 8 |
| 23 | 55 | 114 [59 ; 165.5] | 3 | 0.8 [0.1 ; 1.9] | 2 | 4.6 [2.2 ; 7.4] | 23 | 0 [0 ; 102.3] | 7 |
| 24 | 51 | 115 [57 ; 180] | 1 | 1 [0.1 ; 1.6] | 0 | 4.6 [2.4 ; 9.2] | 22 | 0 [0 ; 90.2] | 7 |
| 25 | 49 | 103 [54 ; 187] | 2 | 0.7 [0.1 ; 1.6] | 3 | 5 [2.9 ; 7.4] | 20 | 0 [0 ; 75.1] | 6 |
| 26 | 44 | 94 [69 ; 171] | 0 | 0.7 [0.1 ; 1.7] | 0 | 3.9 [1.9 ; 7.5] | 16 | 0 [0 ; 62.1] | 2 |
| 27 | 44 | 110 [72 ; 164] | 3 | 0.6 [0.1 ; 1.8] | 1 | 3.6 [1.6 ; 8.8] | 18 | 0 [0 ; 60.1] | 5 |
| 28 | 40 | 101 [62 ; 176] | 5 | 0.8 [0.1 ; 1.4] | 0 | 3.5 [2.3 ; 6.1] | 15 | 0 [0 ; 25.6] | 6 |
| 29 | 39 | 97 [64 ; 187] | 2 | 0.6 [0.1 ; 1.7] | 1 | 3.6 [1.8 ; 7.2] | 17 | 0 [0 ; 39.1] | 5 |
| 30 | 37 | 107 [77 ; 201] | 4 | 0.8 [0.1 ; 1.9] | 0 | 3.6 [1.6 ; 5.9] | 21 | 0 [0 ; 27] | 6 |

Miss: missing; GFR: glomerular filtration rate

Concerning GFR imputation:

- When missing, most of the time another GFR was measured one or two days after allowing for linear imputation.
- Some patients were under dialysis and then remained oliguric during their ICU stay with an increase of their creatinine thereafter. Those patients kept with a GFR at 0 which seemed in accordance with their low renal clearance.

Table S 3: Main characteristics of the population

| **Variable (Number (%) or median (IQR))** | **All (N=312)** | **No ARC at admission (N=246)** | **ARC at admission (N=66)** | **P value** |
| --- | --- | --- | --- | --- |
| Age (years) | 62.7 [51.4 ; 71.8] | 64.3 [53.1; 73] | 55.1 [50.2; 63.5] | <0.01 |
| Gender (male) | 248 (74.7) | 189 (73) | 59 (80.8) | 0.17 |
| Body Mass Index (kg/m²) (NA=44) | 26.9 [24.2 ; 31.7] | 26.8 [24.2 ; 31] | 27.7 [24.7 ; 32.9] | 0.18 |
| **Comorbidities** | |  |  |  |
| Cardiovascular | 106 (31.9) | 96 (37.1) | 10 (13.7) | <.01 |
| Respiratory | 64 (19.3) | 51 (19.7) | 13 (17.8) | 0.72 |
| Hepatic | 16 (4.8) | 13 (5) | 3 (4.1) | 0.75 |
| Diabetes | 40 (12) | 36 (13.9) | 4 (5.5) | 0.05 |
| Immunosuppression | 62 (18.7) | 54 (20.8) | 8 (11) | 0.06 |
| Charlson score | 1 [0 ; 3] | 2 [0 ; 4] | 0 [0 ; 1] | <0.01 |
| Medical reason for admission | 245 (73.8) | 187 (72.2) | 58 (79.5) | 0.21 |
| **Main symptom at admission** | |  |  |  |
| Multiple organ failure | 18 (5.8) | 16 (6.5) | 2 (3) | 0.11 |
| Septic shock | 15 (4.8) | 15 (6.1) | 0 (0) | . |
| Cardiogenic shock | 23 (7.4) | 20 (8.1) | 3 (4.5) | . |
| Other shock | 5 (1.6) | 4 (1.6) | 1 (1.5) | . |
| Acute respiratory distress | 184 (59) | 145 (58.9) | 39 (59.1) | . |
| Acute kidney failure | 2 (0.6) | 2 (0.8) | 0 (0) | . |
| Coma | 15 (4.8) | 11 (4.5) | 4 (6.1) | . |
| Continuous monitoring | 50 (16) | 33 (13.4) | 17 (25.8) | . |
| **Main admission category** | |  |  |  |
| Pneumonia | 172 (51.8) | 132 (51) | 40 (54.8) | 0.56 |
| ARDS | 67 (20.2) | 50 (19.3) | 17 (23.3) | 0.45 |
| Heart failure | 23 (6.9) | 19 (7.3) | 4 (5.5) | 0.58 |
| Cardiorespiratory arrest | 14 (4.2) | 10 (3.9) | 4 (5.5) | 0.54 |
| Endocarditis | 12 (3.6) | 12 (4.6) | 0 (0) | 0.06 |
| SARS COV 2 pneumoniae | 196 (62.8) | 148 (60.2) | 48 (72.7) | 0.06 |
| **Severity on admission** |  |  |  |  |
| SAPS II score | 32 [24 ; 42.5] | 34 [26 ; 45] | 24 [20 ; 34] | <0.01 |
| SOFA score | 5 [3 ; 8] | 5 [3 ; 8] | 4 [3 ; 6] | 0.02 |
| **Treatment on admission** |  |  |  |  |
| Use of vasopressor/inotropes | 146 (44) | 119 (45.9) | 27 (37) | 0.17 |
| Invasive mechanical ventilation | 154 (46.4) | 126 (48.6) | 28 (38.4) | 0.12 |
| Renal replacement therapy | 48 (14.5) | 46 (17.8) | 2 (2.7) | <0.01 |
| Enteral feeding | 98 (29.5) | 82 (31.7) | 16 (21.9) | 0.11 |
| Parenteral feeding | 13 (3.9) | 11 (4.2) | 2 (2.7) | 0.56 |
| Proton pump inhibitors | 225 (67.8) | 185 (71.4) | 40 (54.8) | <0.01 |
| Contrast agent | 112 (33.7) | 83 (32) | 29 (39.7) | 0.22 |
| Anti-microbial therapy at admission | 225 (67.8) | 179 (69.1) | 46 (63) | 0.32 |
| Aminoglycosides | 36 (10.8) | 34 (13.1) | 2 (2.7) | 0.01 |
| **Outcomes** |  |  |  |  |
| ICU Length of stay | 9 [5 ; 17.5] | 9 [5; 18] | 8 [5; 16] | 0.43 |
| ICU mortality | 100 (31) | 90 (35.1) | 10 (16.4) | <0.01 |


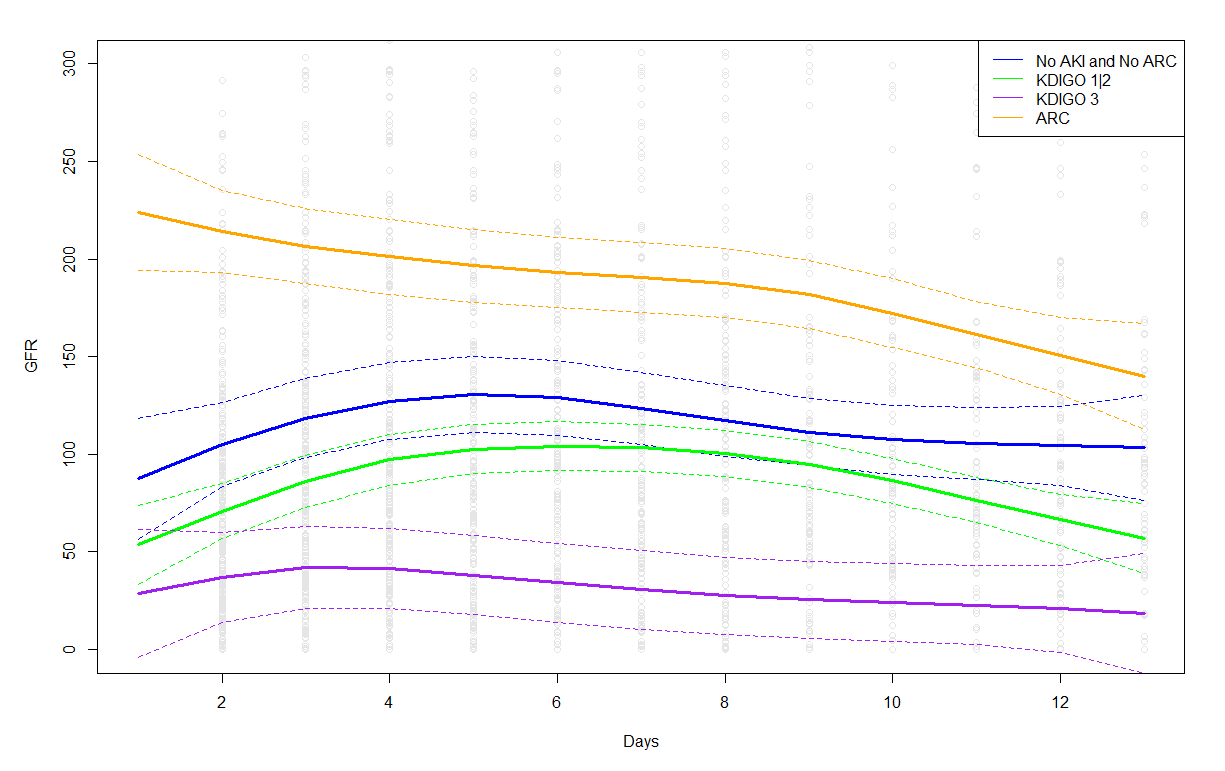


Figure S2: Evolution of Glomerular Filtration Rate (GFR) over time according to the renal status on admission (AKI: Acute Kidney Injury, ARC: Augmented Renal Clearance, KDIGO: Kidney Disease Improve Global Outcome)

Table S 4: ARC occurrence depending on CKD stage using the KDIGO 2012 CKD definition

| CKD | ARC on admission | ARC between Day 3 and 7 | ARC after Day 8 |
| --- | --- | --- | --- |
| Stage 1 | 45/151(29.8%) | 88/151(58.3%) | 49/88(55.6%) |
| Stage 2 | 4/61(6.6%) | 22/61(36.1%) | 8/32(25%) |
| Stage 3 | 0/40 | 5/40(12.5%) | 1/21(4.7%) |
| Stage 4 | 0/13 | 0 | 0 |
| Unknown baseline creatinine | 17/47 (36.1%) | 31/47(66%) | 30/39(76%) |

ARC: Augmented Renal Clearance, CKD: Chronic Kidney Disease, KDIGO: Kidney Disease Improving Global Outcomes

Table S 5: Factors associated with the occurrence of Augmented Renal Clearance on admission (univariate analysis, OR: odd-ratio, CI 95% confidence interval)

| Variable | OR | [OR CI 95%] | P value |
| --- | --- | --- | --- |
| Age <50 | 2.5 | [1.03 - 6.07] | 0.043 |
| Age between 50 et 60 | 5.74 | [2.54 - 12.93] | <0.01 |
| Age between 60 et 70 | 1.59 | [0.67 - 3.79] | 0.30 |
| Age > 70 | 1 |  | <0.01 |
| Sex (Male) | 1.56 | [0.82; 2.97] | 0.18 |
| BMI (kg/m²) |  |  |  |
| BMI > 30 kg/m² | 1.949 | [0.96 ; 3.94] | 0.06 |
| Cardiovascular comorbidities |  |  |  |
| Respiratory comorbidities | 1.571 | [0.89 ; 2.76] | 0.12 |
| Hepatic comorbidities | 0.269 | [0.13 ; 0.57] | <.01 |
| Immunosuppression | 0.870 | [0.42 ; 1.79] | 0.70 |
| Diabetes | 0.853 | [0.24 ; 3.09] | 0.81 |
| Reason for admission | 0.443 | [0.19 ; 1.03] | 0.06 |
| Shock | 0.376 | [0.13 ; 1.1] | 0.07 |
| Acute respiratory failure |  |  |  |
| Other | 0.589 | [0.32 ; 1.1] | 0.10 |
| SARS COV 2 pneumoniae | 1.766 | [0.97 ; 3.21] | 0.06 |
| SAPS II < 24 | 5.81 | [2.28 - 14.77] | <0.01 |
| SAPS II between 24 et 32 | 2.67 | [0.97 - 7.37] | 0.06 |
| SAPS II between 32 et 43 | 1.41 | [0.47 - 4.19] | 0.54 |
| SAPS II > 43 | 1 |  | <0.01 |
| AKI KDIGO 0 | 20.12 | [4.71 - 85.89] | <0.01 |
| AKI KDIGO 1 | 12.5 | [2.73 - 57.21] | <0.01 |
| AKI KDIGO 2 | 4.26 | [0.79 - 22.9] | 0.09 |
| AKI KDIGO 3 | 1 |  | <0.01 |
| Organ support and other treatments on admission* |  |  |  |
| Use of vasopressor/inotropes | 0.739 | [0.43 ; 1.28] | 0.28 |
| Mechanical ventilation | 0.702 | [0.41 ; 1.21] | 0.21 |
| Renal replacement therapy | 0.136 | [0.03 ; 0.58] | <0.01 |
| Enteral nutrition | 0.640 | [0.34 ; 1.19] | 0.16 |
| Parenteral nutrition | 0.738 | [0.16 ; 3.45] | 0.70 |
| Protons pumps inhibitors | 0.518 | [0.3 ; 0.91] | 0.02 |
| Media contrast | 1.447 | [0.83 ; 2.52] | 0.19 |
| Aminoglycoside | 0.195 | [0.05 ; 0.83] | 0.03 |
| Vancomycin | - | - | - |

AKI: Acute Kidney Injury, ARC: Augmented Renal Clearance, BMI: Body Mass Index; ICU: Intensive Care Unit, SOFA: Sequential Organ Failure Assessment, SAPS II: Simplified Acute Physiology Score, IQR: interquartile range, NA: missing data, OR: Odd ratio, IC : Confidence Interval;

*on calendar day 1 or 2

Table S 6: Factors associated with LATE ARC, subdistribution survival analysis with death and living alive as competing risks, univariate analyses

| Variable | HR | HRIC | pvalue |
| --- | --- | --- | --- |
| Age <50 | 0.78 | [0.3 ; 1.98] | 0.60 |
| Age between 50 et 60 | 0.64 | [0.23 ; 1.74] | 0.38 |
| Age between 60 et 70 | 1.30 | [0.68 ; 2.47] | 0.42 |
| Age > 70 | 1 |  | 0.42 |
| Sex (Male) | 1.99 | [0.96 ; 4.12] | 0.06 |
| BMI > 30 kg/m² | 1.21 | [0.68 ; 2.17] | 0.51 |
| Reason for admission |  |  |  |
| Shock | 1.41 | [0.69 ; 2.87] | 0.35 |
| Acute respiratory failure | 0.47 | [0.26 ; 0.87] | 0.02 |
| Other | 1 |  | 0.03 |
| SARS COV 2 pneumoniae | 1.65 | [1.11 ; 2.46] | 0.01 |
| Cardiovascular comorbidities | 0.99 | [0.56 ; 1.75] | 0.97 |
| Respiratory comorbidities | 0.71 | [0.32 ; 1.58] | 0.40 |
| Diabetes | 1.27 | [0.63 ; 2.58] | 0.51 |
| Immunosuppression | 0.86 | [0.44 ; 1.69] | 0.66 |
| Use of vasopressor/inotropes on admission* | 0.90 | [0.51 ; 1.57] | 0.70 |
| Mechanical ventilation on admission* | 0.69 | [0.4 ; 1.22] | 0.20 |
| AKI KDIGO 3 | 0.20 | [0.09 ; 0.42] | <0.01 |
| AKI KDIGO 2 | 0.12 | [0.03 ; 0.5] | <.01 |
| AKI KDIGO 1 | 0.77 | [0.39 ; 1.53] | 0.46 |
| AKI KDIGO 0 | 1 |  | <0.01 |
| Media contrast on admission* | 1.43 | [0.8 ; 2.54] | 0.22 |
| Enteral nutrition on admission* | 1.15 | [0.65 ; 2.04] | 0.63 |
| Parenteral nutrition on admission* | 0.34 | [0.04 ; 2.53] | 0.29 |
| Aminoglycoside on admission* | 0.50 | [0.18 ; 1.38] | 0.18 |
| Vancomycin on admission* | 1.75 | [0.66 ; 4.64] | 0.26 |
| Protons pumps inhibitors on admission* | 1.43 | [0.8 ; 2.54] | 0.22 |

ARC: Augmented Renal Clearance, BMI: Body Mass Index, HR : Hazard ratio, HRIC : 95% confidence interval of the hazard ratio, KDIGO :Kidney Disease Improve Global Outcome, , SOFA: sequential organ failure assessment. *On calendar day 1 or 2

Table S7: Risk factor on admission for death before day 30.

| Variable (N(%)/Median [IQR]) | Alive at ICU discharged | Death in ICU | Pvalue§ |
| --- | --- | --- | --- |
| Patient number | 212 | 100 | . |
| Age | 61.3 [49.6 ; 71.2] | 65.2 [57.9 ; 72.5] | <.01 |
| Gender (Male) | 158 (74.5) | 74 (74) | 0.92 |
| BMI | 27.1 [24.2 ; 32] | 27.1 [24.3 ; 31.1] | 0.68 |
| BMI > 30 kg/m² | 69 (32.5) | 29 (29) | 0.53 |
| Cardiovascular comorbidities | 63 (29.7) | 37 (37) | 0.20 |
| Respiratory comorbidities | 37 (17.5) | 20 (20) | 0.59 |
| Hepatic comorbidities | 11 (5.2) | 5 (5) | 0.94 |
| Immunosuppression | 24 (11.3) | 16 (16) | 0.25 |
| Diabetes | 38 (17.9) | 21 (21) | 0.52 |
| Charlson score | 1 [0 ; 3] | 2 [0 ; 4] | 0.16 |
| Medical reason for admission | 153 (72.2) | 75 (75) | 0.60 |
| Main Reason for admission |  |  | . |
| Shock | 48 (22.6) | 19 (19) | 0.65 |
| Acute respiratory failure | 39 (18.4) | 22 (22) | . |
| Other symptoms | 125 (59) | 59 (59) | . |
| SARS-COV2 pneumoniae | 127 (59.9) | 69 (69) | 0.12 |
| SAPS 2 | 30 [23 ; 40.5] | 38 [28.5 ; 52] | <.01 |
| SOFA score | 4 [2 ; 7] | 7 [3.5 ; 10] | <.01 |
| Use of vasopressor/inotropes* | 90 (42.5) | 56 (56) | 0.03 |
| Mechanical ventilation* | 93 (43.9) | 61 (61) | <.01 |
| Renal replacement therapy* | 21 (9.9) | 27 (27) | <.01 |
| Enteral feeding* | 57 (26.9) | 41 (41) | 0.01 |
| Parenteral feeding* | 6 (2.8) | 6 (6) | 0.17 |
| Protons pumps inhibitor* | 140 (66) | 76 (76) | 0.08 |
| Media contrast* | 72 (34) | 39 (39) | 0.39 |
| Anti-microbial therapy* | 146 (68.9) | 66 (66) | 0.61 |
| Aminoglycoside * | 22 (10.4) | 14 (14) | 0.35 |
| Vancomycin* | 10 (4.7) | 1 (1) | 0.10 |
| AKI KDIGO 3 | 42 (19.8) | 35 (35) | . |
| AKI KDIGO 2 | 30 (14.2) | 19 (19) | . |
| AKI KDIGO 1 | 45 (21.2) | 15 (15) | . |
| AKI KDIGO 0 | 95 (44.8) | 31 (31) | <.01 |
| 1^st^ ARC Day 1-Day 2 | 54 (25.5) | 12 (12) | <.01 |
| 1^st^ ARC between Day 3 and Day 7 | 69 (32.5) | 22 (22) | 0.06 |
| 1^st^ ARC after day 8 | 12 (10.9) | 5 (6.5) | 0.30 |
| % time with CrCl > 130 mL/min/1.37 | 16.7 [0 ; 50] | 0 [0 ; 18.2] | <.01 |
| % time avec CrCl > 200 mL/min/1.73 | 0 [0 ; 20] | 0 [0 ; 4.4] | <.01 |

ARC: Augmented Renal Clearance, BMI: Body Mass Index, CrCl : Creatinine Clearance , AKI: Acute Kidney Injury, ICU : Intensive Care Unit, , IQR: interquartile range, KDIGO :Kidney Disease Improve Global Outcome, SAPS II: Simplified Acute Physiology Score, SOFA: sequential organ failure assessment.

*on calendar 1 or 2;

§Fisher exact test or Wilcoxon test

Table S8: Probability of occupying a state at a given time (Augmented renal clearance, no acute kidney injury, acute kidney injury KDIGO 1-2 or acute kidney injury KDIGO 3)

| Day |  | ARC | No ARC/No AKI | AKI KDIGO 1 or 2 | AKI KDIGO 3 |
| --- | --- | --- | --- | --- | --- |
|  |  | Probability of occupancy (sd) | | | |
| Day 5 | ARC | 0.12(0.02) | 0.13(0.02) | 0.12(0.02) | 0.06(0.02) |
|  | AKI KDIGO 0 | 0.16(0.03) | 0.18(0.03) | 0.17(0.03) | 0.09(0.02) |
|  | AKI KDIGO 1 or 2 | 0.15(0.03) | 0.19(0.03) | 0.22(0.03) | 0.19(0.04) |
|  | AKI KDIGO 3 | 0.02(0.01) | 0.03(0.01) | 0.06(0.02) | 0.13(0.04) |
|  | Discharge alive from ICU | 0.52(0.07) | 0.4(0.04) | 0.35(0.04) | 0.32(0.08) |
|  | Death | 0.04(0.02) | 0.06(0.02) | 0.08(0.02) | 0.21(0.07) |
| Day 10 | ARC | 0.04(0.01) | 0.04(0.01) | 0.04(0.01) | 0.06(0.02) |
|  | AKI KDIGO 0 | 0.08(0.02) | 0.1(0.02) | 0.11(0.02) | 0.04(0.01) |
|  | AKI KDIGO 1 or 2 | 0.08(0.02) | 0.09(0.02) | 0.1(0.02) | 0.08(0.02) |
|  | AKI KDIGO 3 | 0.02(0.01) | 0.02(0.01) | 0.03(0.01) | 0.09(0.02) |
|  | Discharge alive from ICU | 0.7(0.05) | 0.61(0.04) | 0.56(0.04) | 0.03(0.01) |
|  | Death | 0.09(0.02) | 0.12(0.03) | 0.16(0.03) | 0.47(0.07) |
| Day 15 | ARC | 0.03(0.01) | 0.04(0.01) | 0.04(0.01) | 0.03(0.01) |
|  | AKI KDIGO 0 | 0.03(0.01) | 0.03(0.01) | 0.03(0.01) | 0.03(0.01) |
|  | AKI KDIGO 1 or 2 | 0.04(0.01) | 0.05(0.01) | 0.05(0.01) | 0.04(0.01) |
|  | AKI KDIGO 3 | 0.02(0.01) | 0.03(0.01) | 0.03(0.01) | 0.03(0.01) |
|  | Discharge alive from ICU | 0.75(0.04) | 0.67(0.03) | 0.63(0.03) | 0.52(0.07) |
|  | Death | 0.13(0.03) | 0.18(0.03) | 0.22(0.03) | 0.35(0.07) |
| Day 30 | ARC | 0.01(0) | 0.01(0.01) | 0.01(0.01) | 0.01(0.01) |
|  | AKI KDIGO 0 | 0(0) | 0(0) | 0(0) | 0(0) |
|  | AKI KDIGO 1 or 2 | 0.01(0.01) | 0.01(0.01) | 0.01(0.01) | 0.01(0.01) |
|  | AKI KDIGO 3 | 0(0) | 0(0) | 0(0) | 0(0) |
|  | Discharge alive from ICU | 0.79(0.04) | 0.73(0.03) | 0.68(0.03) | 0.57(0.07) |
|  | Death | 0.19(0.03) | 0.25(0.03) | 0.3(0.03) | 0.41(0.07) |

AKI: Acute Kidney Injury, ARC: Augmented Renal Clearance, ICU: Intensive Care Unit, KDIGO : Kidney Disease Improving Global Outcomes, sd : standard deviation

Table S 9: Impact of ARC on death, Multivariable Cause Specific Cox model with ARC considered as a time dependent variable, Landmark approach from day 2 to day 7*

|  | Risk of death | | | Risk of leaving alive from ICU | | |
| --- | --- | --- | --- | --- | --- | --- |
| Variable | csHR | csHRIC | pvalue | csHR | csHR CI 95% | Pval |
| Day 2 | 0.17 | [0.06 ; 0.46] | <.01 | 1.05 | [0.76 ; 1.45] | 0.78 |
| Day 3 | 0.17 | [0.06 ; 0.47] | <.01 | 1.14 | [0.82 ; 1.59] | 0.43 |
| Day 4 | 0.20 | [0.07 ; 0.54] | <.01 | 1.12 | [0.78 ; 1.6] | 0.55 |
| Day 5 | 0.21 | [0.08 ; 0.57] | <.01 | 1.35 | [0.92 ; 1.99] | 0.13 |
| Day 6 | 0.17 | [0.05 ; 0.54] | <.01 | 1.45 | [0.96 ; 2.19] | 0.08 |
| Day 7 | 0.20 | [0.06 ; 0.64] | 0.01 | 1.44 | [0.91 ; 2.27] | 0.12 |

ARC: Augmented Renal Clearance, cs: Cause specific, HR: Hazard ratio, HRIC : 95% confidence interval of the hazard ratio*Adjustment on age and invasive mechanical ventilation on admission

Table S 10: Impact of ARC on death, Multivariate Sub distribution survival model considering leaving alive from ICU as a competing risk, using a landmark approach from day 2 to day 7*

|  | Risk of death | | |
| --- | --- | --- | --- |
| Variable | SubHR | SubHR CI 95% | pvalue |
| Day 2 | 0.56 | [0.29 ; 1.1] | 0.09 |
| Day 3 | 0.84 | [0.47 ; 1.5] | 0.56 |
| Day 4 | 0.42 | [0.21 ; 0.83] | 0.01 |
| Day 5 | 0.46 | [0.22 ; 0.97] | 0.04 |
| Day 6 | 0.63 | [0.33 ; 1.22] | 0.17 |
| Day 7 | 0.44 | [0.22 ; 0.9] | 0.02 |

ARC: Augmented Renal Clearance, HR: Hazard ratio, HRIC: 95% confidence interval of the hazard ratio

*Adjustment on age, invasive mechanical ventilation, and renal replacement therapy on admission
